# Multi-Scale Compositionality: Identifying the Compositional Structures of Social Dynamics Using Deep Learning

**DOI:** 10.1371/journal.pone.0118309

**Published:** 2015-04-01

**Authors:** Huan-Kai Peng, Radu Marculescu

**Affiliations:** 1 Department of Electrical and Computer Engineering, Carnegie Mellon University, Pittsburgh, Pennsylvania, USA; Universitat Rovira i Virgili, SPAIN

## Abstract

**Objective:**

Social media exhibit rich yet distinct temporal dynamics which cover a wide range of different scales. In order to study this complex dynamics, two fundamental questions revolve around (1) the signatures of social dynamics at different time scales, and (2) the way in which these signatures interact and form higher-level meanings.

**Method:**

In this paper, we propose the Recursive Convolutional Bayesian Model (RCBM) to address both of these fundamental questions. The key idea behind our approach consists of constructing a deep-learning framework using specialized convolution operators that are designed to exploit the inherent heterogeneity of social dynamics. RCBM’s runtime and convergence properties are guaranteed by formal analyses.

**Results:**

Experimental results show that the proposed method outperforms the state-of-the-art approaches both in terms of solution quality and computational efficiency. Indeed, by applying the proposed method on two social network datasets, Twitter and Yelp, we are able to identify the compositional structures that can accurately characterize the complex social dynamics from these two social media. We further show that identifying these patterns can enable new applications such as anomaly detection and improved social dynamics forecasting. Finally, our analysis offers new insights on understanding and engineering social media dynamics, with direct applications to opinion spreading and online content promotion.

## Introduction

All activities in social networks evolve over time. Consequently, understanding the structures behind social dynamics represents a central question in social networks research, with many important applications including political campaigning [1], viral marketing [2], and disaster response [3]. While several recent works have investigated methods to identify patterns of social dynamics [4–7], in this paper, we study a new, unexplored perspective of social dynamics, namely, *multi-scale compositionality*.

Studying multi-scale compositionality consists of identifying the *compositional structures* of social media dynamics, which generally covers two tasks:

T1. *Identification of multi-scale signatures*, which consists of identifying distinct signatures across a range of time scales, as opposed to sticking with a single one;
T2. *Mining of compositional interactions*, which requires discovering the interaction among multiple such signatures that produce higher-level meanings.

To illustrate these ideas, consider the case of human face recognition, where the first task includes recognizing the eyebrows, the cheeks, or the overall head shape. In contrast, the second task includes gauging the distance between the eyebrows, measuring the angle between the jaw and the ears, or recognizing the polygon formed by the lips, cheeks, and eyebrows. To recognize a human face, both tasks are equally important: one could make a mistake by either recognizing the wrong shape of an eyebrow, or by over/underestimating the distance between the eyebrows.

In the context of social dynamics, we find the same two tasks as being equally relevant. Indeed, social media exhibit distinct signatures at various time scales that range from seconds to days, whereas different combinations of such signatures can have totally different meanings and consequences. For example, an intense popularity of some keywords followed by a vibrant discussion may indicate a trendy event; however, the same popularity without any follow-up discussion can, on the contrary, indicate an internet scam. Clearly, being able to distinguish between the two cases can make a big difference.

In this paper, we propose a new model, namely, the Recursive Convolutional Bayesian Model (RCBM), which is capable of addressing both tasks. The idea of RCBM is building a layered structure of signature detectors, where each layer is responsible for a specific time scale. Moreover, a higher-level layer is capable of detecting the interactions of various signatures (as they come from its immediate lower layer), and hence can identify compositional structures.

To the best of our knowledge, this work brings the following new contributions:

1. *Design and Analysis of RCBM*: We propose RCBM, a new deep learning framework based on specialized convolution operators. While the formulation of RCBM is general enough to consider the heterogeneity of social signals, its runtime performance and solution quality are analyzed formally and confirmed experimentally. Of note, this is the first time when deep learning is used in the context of social dynamics.
2. *Identifying the Compositional Structures of Social Dynamics*: Using RCBM, we discover that the social dynamics in Twitter are characterized by signatures representing the dynamics’ popularity, contagiousness, stickiness, and interactivity. On the other hand, the social dynamics in Yelp are characterized by signatures representing how different groups of reviewers rate individual businesses. Further, we find the patterns where theses signatures interact by generating, enhancing, or dominating one another.
3. *RCBM-Enabled Applications*: We investigate new applications enabled by RCBM, including the detection of abnormal social dynamics and the forecasting of social dynamics with features extracted using RCBM.

Finally, our RCBM belongs to a broader family of methods called *deep learning*[8, 9], which has recently revolutionized the fields of Computer Vision [10–12] and Natural Language Processing [13, 14] by generating results that outperform the previous state-of-the-arts both in unsupervised and supervised settings. Being the first work that introduces deep learning to the social realm, we believe our paper can serve as a starting point toward defining many new research directions and applications.

### Related Work

There are three main lines of research that are relevant to the topic presented here: (1) mining patterns of social dynamics, (2) time series dictionary learning, and (3) deep learning.

#### Mining patterns of social dynamics

Several papers have investigated methods of identifying patterns for social dynamics [4–7]. In particular, endogenous vs. exogenous trends are studied in [4]; the shape of aggregate popularity is looked at in [5]; the proportions of readership before, at, and after the peak are investigated in [6]; an efficient clustering algorithm for multi-dimensional social dynamics in general is proposed in [7]. Our work compliments all these works by additionally considering the tasks T1 and T2 mentioned in Section 1.

#### Time-series dictionary learning

The research in time-series dictionary learning targets the general problem of mining structures from time-series streams [15–19]. Along this line, the authors of [15, 16] make use of a fixed-length sliding window to extract the subsequences followed by conventional clustering methods. Since the length of the sliding window is fixed, neither task T1 nor T2 above is addressed. In [17], a method based on *minimum description length* is proposed to consider variable scales. Also, the authors of [18, 19] introduce the concept of *shapelets* that does not rely on specific scales. Although T1 is partially addressed in these works, T2 is not considered. Moreover, latent factor methods like [20, 21] model multivariate time series using hidden variables. Along this line, the State Space Model [20] builds a linear dynamical system assuming time-invariance and linearity. Also, Sparse Coding [21] can be used to discover global signatures for pre-aligned time series. Although these methods also make use of latent factors like RCBM, they do not address T1 and T2. Finally, the authors of [22, 23] combine a convolutional formation with sparse coding. While the work of [22] does not address T1 and T2, the work of [23] is designed for image applications where the input matrix is assumed to be homogeneous in both dimensions. Our method here is different, since it is designed for social dynamics where the input matrix is assumed to be homogeneous in one direction (i.e., time) but heterogeneous in another (i.e., feature).

#### Deep learning

Research in deep learning has recently gained much attention in supervised learning such as classification and regression [13, 14], as well as unsupervised learning such as feature extraction [11] and dimension reduction [24]. Moreover, there are also works that formulate deep learning using convolution [10, 12]. In particular, the authors of [12] propose the Convolutional Deep Belief Network (cDBN), which combines the Reduced Boltzmann Machine with the matrix convolution. Its sampling-based learning algorithm, however, is not efficient enough for practical use. The Convolutional Autoencoder (cAE) proposed in [10] represents the current state-of-the-art among convolutional deep architectures. When applied to image recognition, it not only produces meaningful features that mimic the ones used by human’s visual cortex area V2, but it also generates classification results that outperform the state-of-the-art. When applied to social dynamics, our proposed RCBM has two advantages over cAE. First, cAE uses the conventional convolution operator that overlooks the heterogeneity inherent in social dynamics. RCBM, in contrast, uses specialized convolution operators that exploit the heterogeneity of social dynamics, and therefore offers higher-quality solutions yet requires much less runtime. Second, the higher-level layers of cAE have much more hidden variables compared to its lowest-level layer. In RCBM, on the contrary, the number of hidden variables of each layer remains roughly the same, which further enhances RCBM’s efficiency.

## Materials and Methods

We now present our Recursive Convolutional Bayesian Model (RCBM), a probabilistic model that learns the compositional structures of social dynamics. In turn, we introduce the base Convolutional Bayesian Model (CBM), its learning algorithm, and the way to construct an RCBM using multiple-layer CBM’s.

### CBM: the Base Model

#### Problem definition

**Fig 1:**
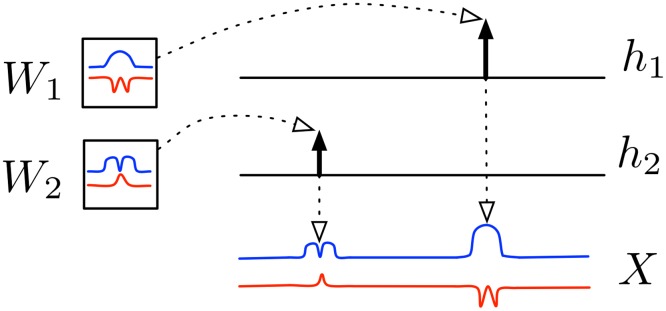
CBM’s generation process. *W*: filter matrices; *h*: activation vectors; *X*: social dynamic. The filters *W*_1_ and *W*_2_ are activated differently depending on their corresponding activation vectors *h*_1_ and *h*_2_, respectively.

We use a generic *information token* (e.g., Youtube video, photo, hashtag, etc) as the proxy of social dynamics. Since the social dynamics that emerge while an information token is being propagated across a social network can be characterized by multiple statistics (e.g., the ones mentioned in Section 2), we use *X* ∈ *R* ^*D*×*T*^ to represent the *D*-dimensional social dynamics corresponding to an information token (e.g., *D* = 2 for the *X* in Fig. 1). The precise definition of *X* depends on the dataset and the application. See Section 5.1 for the cases of using Twitter and Yelp datasets. All notations in this paper are summarized in Table 1. Given a set of social dynamics ${\left\{X^{\left(i\right)}\right\}}_{i=1}^{\left(n\right)}$ (associated with *n* information tokens), our problem is defined as finding a set of *D*-dimensional *structures* (e.g., the *W*_1_ and *W*_2_ in Fig. 1) that best characterize these dynamics.

**Table 1:** Summary of all notations in this paper.

Base Model	
*X*	social-dynamic matrix. *X* ∈ *R* ^*D*×*T*^
*W*_*k*_	the *k*-th filter matrix. $W_{k}\in R^{D\times T_{w}}$
*h*_*k*_	the *k*-th activation vector. $h_{k}\in R_{+}^{T}{}^{+T_{w}-1}$
*σ*, *β*	parameters of *P*(*X*|*h*) and *P*(*h*), respectively
*K*	the number of filters (in one level)
*T*_*w*_	filter scale
Model Learning	
${\left\{X^{\left(i\right)}\right\}}_{i=1}^{\left(n\right)}$	set of *n* sample social dynamics
*n*	size of sample social dynamics
$h_{k}^{\left(i\right)}$	the *k*-th activation vector of the *i*-th sample
*t*_*h*_*k*__,*t*_*W*_*k*__	step-sizes for updating *h*_*k*_ and *W*_*k*_, respectively
$h_{k}^{\left[r\right]}$	the solution of *h*_*k*_ in the *r*-th optimization iteration
$W_{k}^{\left[r\right]}$	the solution of *W*_*k*_ in the *r*-th optimization iteration
Stacking Multiple Layers	
*X*_*l*_	input dynamic at level *l*
*h*_*l*,*k*_	the *k*-th activation vector at level *l*
*W*_*l*,*k*_	the *k*-th filter matrix at level *l*
*K*_*l*_	the number of filters at level *l*
*c*	the factor used for downsampling
*L*	the number of levels of an RCBM

#### Assumptions

We aim at solving the above problem under the following three assumptions:

A1 *Finite Structures*: the social dynamics can be characterized by a finite number of structures that are invariant to shifting in time and scaling in magnitude.
A2 *Burstiness*: the distribution for the magnitude of the social dynamics is right-skewed; it is typically small but can be occasionally very large.
A3 *Heterogeniety*: for each *D*-dimensional structure, all dimensions have different meanings and no one is an exact copy of another.

The validity of these assumptions has been reported by many previous authors. Indeed, A1 is discussed in [5–7], A2 in [1, 4, 25], and A3 in [5, 26].

#### Model

We postulate that each social dynamic *X* is generated by *random activations of filters*. For illustration, consider Fig. 1 where *W*_1_ and *W*_2_ represent two *filter matrices*, while *h*_1_ and *h*_2_ represent their *activation vectors*, respectively. From the figure, the social dynamic *X* is generated by making *copies* of the filter matrices *W*_1_ and *W*_2_. Moreover, the activation vectors determine the time-shift and the magnitude of these two copies: *h*_2_ is active earlier but weaker, hence the first weaker signal in *X*; *h*_1_ is active later but stronger, hence the latter stronger signal.

Formally, given a set of *K* filters ${\left\{W_{k}\right\}}_{k=1}^{K}$, our generation process for a social dynamic *X* is:

$$\begin{array}{c} \begin{array}{cc} 1. & \text{Sample}\;{\left\{h_{k}\right\}}_{k=1}^{K}\;\text{such}\;\text{that}\;h_{k}\left[t\right]\;\;∼Exp\left(\beta \right)\;\;\forall k,t\\ 2. & X=\sum_{k}W_{k}⊗h_{k}\;+\;\epsilon \;\text{where}\;\epsilon ∼N\left(0,\sigma^{2}\right). \end{array} \end{array}$$ (1)

Here *Exp*(⋅) and *N*(⋅) denote the Normal and Exponential distributions, respectively, with parameters *β* and *σ*. Also, ⊗ denotes our specialized convolutional operator that carries out the “scale-and-copy” task illustrated in Fig. 1. It is defined as:

$$\begin{array}{c} \left(W⊗h\right)\left[d,t\right]=\sum_{s=1}^{T_{w}}h\left[t+T_{w}-s\right]\cdot W\left[d,s\right]\;\;\;\forall d,t. \end{array}$$ (2)

Note that the ⊗ operator differs from the conventional matrix convolution used in [10, 12]. Effectively, ⊗ does *D* 1-D convolutions between each row of *W* and the entire *h*, and puts the results back to each row of the output matrix separately. Moreover, the above generation process implies a joint distribution *P*(*X*,*h*) = *P*(*X*|*h*)*P*(*h*) where:

$$\begin{array}{c} \begin{array}{ccc} P(X|h;W,\sigma ) & = & \frac{\sqrt{2}}{\sigma \sqrt{\pi }}\exp\left(\frac{||X-\sum_{k}W_{k}⊗h_{k}{\left|\right|}_{F}^{2}}{-2\sigma^{2}}\right)\\ P(h;\beta ) & = & \frac{1}{\beta }\exp\left(\frac{\sum_{k}\left|\right|h_{k}{\left|\right|}_{1}}{-\beta }\right). \end{array} \end{array}$$ (3)

#### CBM features

The design of CBM closely reflects our assumptions A1 ∼ A3. To address A1, we use a convolutional formulation such that the structures (i.e., the filters *W*’s) are invariant to shifting in time and scaling in magnitude. To address A2, we enforce burstiness by assuming that the magnitude of the activation vectors (i.e., *h*’s) follows an exponential distribution, which is typically small but occasionally large. This will also enforce sparsity for activation vectors during model learning (see Section 3.2). Finally, to address A3, we consider heterogeneity using our specialized convolutional operator ⊗ instead of the conventional matrix convolution. As we will show in Section 4, this provides provable advantages in both runtime and solution quality.

### CBM Model Learning

Since given *W* and *h*, the Maximum Likelihood Estimators (MLE) for *σ* and *β* (in Equation 3) can be calculated in closed form, the main challenge for learning a CBM lies in estimating *W* in presence of the hidden variables *h*_*k*_’s. Formally, the problem can be written as:

$$\begin{array}{c} W^{*}=\underset{W}{arg max}\log P\left(X\right)=\underset{W}{arg max}\;\;log\int P\left(X|h\right)P\left(h\right)dh. \end{array}$$ (4)

Assuming that *P*(*W*,*h*) peaks with respect to (w.r.t.) *h*, we obtain the approximation:

$$\begin{array}{c} \begin{array}{ccc} W^{*} & \approx & {\text{arg max}}_{W}\;max_{h}\log P(X|h)P\left(h\right)\\ & = & {\text{arg max}}_{W,h}\frac{-1}{2}{\left\|X-\sum_{k}W_{k}⊗h_{k}\right\|}_{F}^{2}-\frac{\sigma^{2}}{\beta }\sum_{k}{\left\|h_{k}\right\|}_{1}, \end{array} \end{array}$$ (5)

where ||⋅||_*F*_ denotes the Frobenius norm. Now, considering a set of *n* samples of social dynamics ${\left\{X^{\left(i\right)}\right\}}_{i=1}^{n}$ and their corresponding activation vectors ${\left\{{\left\{h_{k}^{\left(i\right)}\right\}}_{k=1}^{K}\right\}}_{i=1}^{n}$, Equation 5 becomes:

$$\begin{array}{c} \begin{array}{cc} {\text{arg min}}_{W,h} & \underset{i}{\sum }\left(\frac{1}{2}||X^{\left(i\right)}-\underset{k}{\sum }W_{k}⊗h_{k}^{\left(i\right)}|{|}_{F}^{2}+\frac{\sigma^{2}}{\beta }\underset{k}{\sum }||h_{k}^{\left(i\right)}|{|}_{1}\right)\\ s.t. & ||W_{k}|{|}_{F}\leq 1\forall k\\ & h_{k}^{\left(i\right)}\geq 0\forall k,i. \end{array} \end{array}$$ (6)

In Equation 6, two additional constraints are incorporated to improve the solution quality of *W*. Specifically, the first constraint prevents *W*_*k*_ from blowing up, because otherwise the objective function can be trivially improved by scaling up (and down) *W*_*k*_ (and *h*_*k*_) by the same factor. Also, the second constraint helps ensure that the signs of *W*_*k*_ are not arbitrary and hence can be interpreted coherently. We note that Equation 6 is similar to *sparse coding* in [21] with two important distinctions. First, the conventional matrix multiplication is used in sparse coding whereas a convolutional formulation is used in Equation 6. Second, in sparse coding, the penalty strength (usually denoted as *λ*) needs to be tuned manually, whereas in Equation 6, the value of $\frac{\sigma^{2}}{\beta }$ can be assigned using MLE with a straightforward meaning.

To solve Equation 6, since the problem is convex w.r.t. each one of *W* and *h* (but not both), we alternate between optimizing over one of them while keeping the other one fixed. To start with, we first derive the derivatives of the smooth part of the objective function (i.e., $f_{1}(W,h)=\frac{1}{2}{\left\|X^{\left(i\right)}-\sum_{k}W_{k}⊗h_{k}^{\left(i\right)}\right\|}_{F}^{2}$) w.r.t. *h* and *W*:

$$\begin{array}{c} \begin{array}{ccc} \nabla f_{1}\left(h_{k}^{\left(i\right)}\right) & = & \tilde{W_{k}}⊙\left(\sum_{j}h_{j}^{\left(i\right)}⊗W_{j}\;-\;X\right)\\ \nabla f_{1}\left(W_{k}\right) & = & \sum_{i}\tilde{h_{k}^{\left(i\right)}}⊗\left(\sum_{j}h_{j}^{\left(i\right)}⊗W_{j}\;-\;X\right). \end{array} \end{array}$$ (7)

Here, the deconvolution operator ⊙ is defined as:

$$\begin{array}{c} \left(W⊙X\right)\left[t\right]=\sum_{d=1}^{D}\sum_{s=1}^{T_{w}}X\left[d,t-s+1\right]\cdot W\left[d,s\right]. \end{array}$$ (8)

Again, the ⊙ operator differs from the conventional matrix convolution. Effectively, it calculates the 1-D convolutions of individual rows of *W* and *X* separately, and then adds them together to form a single row. This brings the same advantages as ⊗ does as mentioned at the end of Section 3.1.

#### Stepsize assignment

Typically, one can use line search [27] to determine the stepsize in gradient-based methods. In our case, however, doing so would slow down the optimization considerably because the line search itself needs many additional convolutions. Therefore, we derive the following stepsize assignments for *h* and *W*, respectively:

$$\begin{array}{c} \begin{array}{ccc} t_{h_{k}} & = & \frac{\alpha }{\left|\right|W_{k}{\left|\right|}_{1}^{2}}\\ t_{W_{k}} & = & \frac{\alpha }{\sum_{i}\left|\right|h_{k}^{\left(i\right)}{\left|\right|}_{1}^{2}}, \end{array} \end{array}$$ (9)

where *α* ∈ (0,2). In Section 4, we show that these stepsize assignments are essential to ensure good runtime and convergence properties.

#### Overall algorithm

Algorithm 1 provides the pseudocode for CBM learning. It takes as inputs a set of *n* sample social dynamics ${\left\{X^{\left(i\right)}\right\}}_{i=1}^{n}$, the scale of the filters *T*_*w*_, and the number of filters *K*, and produces as outputs all model parameters including ${\left\{W_{k}^{\left[r\right]}\right\}}_{k=1}^{K}$, *σ*, and *β*. In each iteration of the main repeat loop of Algorithm 1, three tasks are executed in turn: Task 1 (the first for-loop) consists of solving Equation 6 w.r.t. *h*; Task 2 (the second loop) consists of advancing one step toward the solution of Equation 6 w.r.t. *W*; Task 3 (the reminder two lines) consists of calculating the MLE for *σ* and *β*.

The details of Task 1 are presented in Algorithm 2. This is basically designed based on the Nestrov acceleration [28] and the proximal method [27], where the function $S_{\lambda }^{+}(\cdot )$ is an element-wise function defined as:

$$\begin{array}{c} S_{\lambda }^{+}\left(u\right)=\left\{\begin{array}{cc} u-\lambda & \text{if}\;u>\lambda \\ 0, & \text{otherwise}. \end{array}\right. \end{array}$$

Task 2 is conceptually similar to Task 1, where Π(⋅) is defined as:

$$\begin{array}{c} \Pi \left(W\right)=\left\{\begin{array}{cc} W/{\left||W|\right|}_{F} & {\text{if}\;||W||}_{F}>1\\ W, & \text{otherwise}. \end{array}\right. \end{array}$$

**Algorithm 1** Learning of CBM

**Data:** ${\left\{X^{\left(i\right)}\right\}}_{i=1}^{n}$: *n* sample social dynamics

**Data:** *T*_*w*_: scale of the filters

**Data:** *K*: number of filters

**Result:** ${\left\{W_{k}\right\}}_{k=1}^{K}$: solution filters

**Result:** *σ*, *β*: additional model parameters

$W_{k}^{\left[-1\right]}=W_{k}^{\left[0\right]}=$ random initialization ∀*k*

*σ* = *β* = 1

*λ* = *σ* ^2^ *t*_*h*_*k*__/*β*

*r* = 0

**repeat**

*r* = *r* + 1

**for** *i* = 1 **to** *n* **do**

**for** *k* = 1 **to *K* do**

${\left\{h_{k}^{\left(i\right)}\right\}}_{k=1}^{K}$ = optimize_over_h $\left(X^{\left(i\right)},{\left\{W_{k}^{\left[r-1\right]}\right\}}_{k=1}^{K},\sigma ,\beta \right)$

**end**

**end**

**for** *k* = 1 **to** *K* **do**

$t_{W_{k}}=\alpha /\sum_{i}||h_{k}^{\left(i\right)}|{|}_{1}^{2}$

$y=W_{k}^{\left[r-1\right]}+\frac{r-2}{r+1}(W_{k}^{\left[r-1\right]}-W_{k}^{\left[r-2\right]})$

$W_{k}^{\left[r\right]}=\Pi (y-t_{h_{k}}\nabla f_{1}(y))$

**end**

$\sigma ={\left(\frac{1}{n}\sum_{i}||X^{\left(i\right)}-\sum_{k}W_{k}^{\left[r\right]}⊗h_{k}^{\left(i\right)}|{|}_{F}^{2}\right)}^{\frac{1}{2}}$

$\beta =\frac{1}{n}\underset{i,k}{\sum }{\left\|h_{k}^{\left(i\right)}\right\|}_{1}$

**until** *convergence*;

return ${\left\{W_{k}^{\left[r\right]}\right\}}_{k=1}^{K}$, σ, and β

One distinction is that instead of solving *h* until convergence as in Task 1, only a single update is conducted here. Finally, Task 3 calculates the close-form solution of MLE for *σ* and *β*. Since the whole algorithm can be viewed as a case of Coordinate Descent [27], it is guaranteed to converge.

#### Specifying parameters

Algorithm 1 has two parameters, *T*_*w*_ and *K*, that need to be supplied by the user. The filter scale *T*_*w*_ can be conveniently specified as any small number (e.g., letting *T*_*w*_ ≈ *D*) without the need to worry about overlooking the structures at larger scales. This is because the high-level structures with larger scales are meant to be captured by the CBM’s at higher levels (that will be described later).

Regarding the number of filters *K*, since CBM has a natural corresponding probabilistic model (i.e., *P*(*X*,*h*) according to Equation 3), a naive method is trying out a range of different *K*’s and select the one that produces the highest Bayesian Information Criterion (BIC) [29], where the latter is a standard metric for model selection. Doing so, however, is very expensive because it requires training a large number of CBM’s. Therefore, we propose the following three-step method for selecting *K*:

1. Pick a large *K* and train a CBM.
2. Sort all filters such that:
  $$\begin{array}{c} p\leq q\Leftrightarrow \sum_{i=1}^{n}\sum_{k=1}^{p}\left|\right|h_{k}^{\left(i\right)}{\left|\right|}_{1}\geq \sum_{i}\sum_{k=1}^{q}\left|\right|h_{k}^{\left(i\right)}{\left|\right|}_{1}. \end{array}$$ (10)
**Algorithm 2** Optimization over *h*
**Data:** *X*: a sample social dynamic
**Data:** ${\left\{W_{k}\right\}}_{k=1}^{K}$: filter matrices
**Data:** σ, β: model parameters
**Result:** ${\left\{h_{k}\right\}}_{k=1}^{K}$: solution activation vectors
$h_{k}^{\left[-1\right]}=h_{k}^{\left[0\right]}$ = random initialization ∀*k*
$t_{h_{k}}=\alpha /\left({\left\|W_{k}\right\|}_{1}^{2}\right)$ ∀*k*
$\lambda =\sigma^{2}t_{h_{k}}/\beta$
*r* = 0
**repeat**
   
*r* = *r* + 1
   
**for** *k* = 1 **to** *K* **do**
    
$y=h_{k}^{\left[r-1\right]}+\frac{r-2}{r+1}\left(h_{k}^{\left[r-1\right]}-h_{k}^{\left[r-2\right]}\right)$
    
$h_{k}^{\left[r\right]}=S_{\lambda }^{+}\left(y-t_{h_{k}}\nabla f_{1}\left(y\right)\right)$
   
**end**
**until** *convergence*;
  return ${\left\{h_{k}^{\left[r\right]}\right\}}_{k=1}^{K}$
3. Plot the the *cumulative activation function* *F*(*m*):
  $$\begin{array}{c} F\left(m\right)=\sum_{i=1}^{n}\sum_{k=1}^{m}\left|\right|h_{k}^{\left(i\right)}{\left|\right|}_{1} \end{array}$$ (11)
  and pick the new *K* as the position *m* ^*^ such that *F*(*m* ^*^) starts to saturate (i.e., when $\frac{dF}{dm}\leq \varepsilon$ where 0 < *ε* ≪ 1 is a small positive number).

The idea behind our method is that, since sparsity is enforced on *h*_*k*_’s using the one-norm in Equation 6, the irrelevant filters {*W*_m^*^+1_,…,*W*_*K*_} will all have very low activations compared to that of the relevant filters {*W*_1_,…,*W*_m^*^_}. The advantage of this approach is that it requires training only *one* (instead of a large number of) CBM, and hence it is much more efficient. The effectiveness of this method is validated in Section 5.

### RCBM: recursive CBM’s

**Fig 2:**
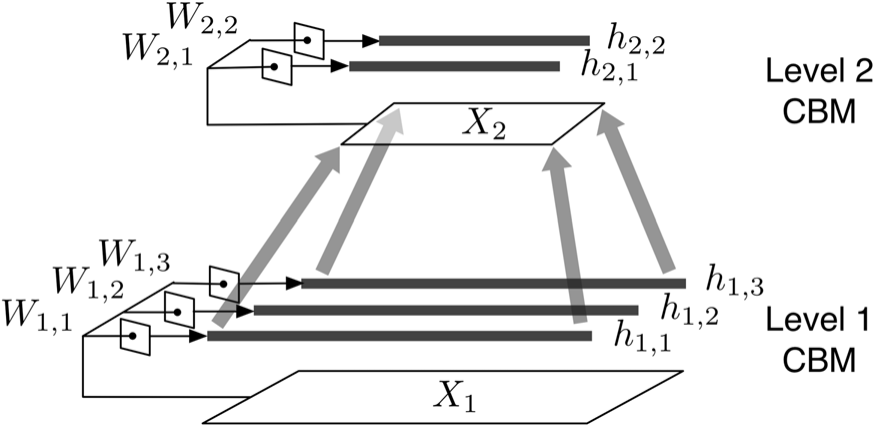
A two-level RCBM. *W*: filter matrices; *h*: activation vectors; *X*: input dynamics. The upper-level input dynamic is constructed by max-pooling the activation vectors that are one level below it.

To capture the compositional structure of social dynamics across different scales, we now introduce RCBM, a hierarchical architecture constructed by stacking together multiple CBM’s, as illustrated in Fig. 2. For some new notations, we use *l* to represent any variable at the *l*-th level, including *X*_*l*_ (input dynamic of level *l*), *h*_*l*,*k*_ (the *k*-th activation vector at level *l*), *W*_*l*,*k*_ (the *k*-th filter matrix in level *l*) and *K*_*l*_ (the number of filters in level *l*). Also, *L* denotes the total number of levels of an RCBM.

Suppose we have trained a CBM with *K* = 3 following the procedures described in Section 3.2, like the Level 1 CBM in Fig. 2. To raise the level of abstraction, we construct the input dynamics at level 2 (i.e., *X*_2_) by down-sampling the lower-level activation vectors (i.e., *h*_1,1_, *h*_2,1_, and *h*_3,1_) by a factor of *c* using a non-linear *max-pooling*[10, 12], which simply takes the maximum value among consecutive *c* values. For example, since *K*_1_ = 3 in Fig. 2, *X*_2_ will have three rows of length $\left\lceil \frac{T+T_{w}-1}{3}\right\rceil$, where *T*+*T*_*w*_−1 is the length of *h*_*k*,1_. Moreover, the values of *X*_2_ will be assigned as *X*_2_[*d*,*t*] = max_*s* ∈ {1,…,*c*}_ *h*_*d*,1_[*c*(*t*−1)+*s*].

After doing max-pooling for each sample, we obtain a set of level-2 dynamics (i.e., *X*_2_) for the whole dataset. We can then use these level-2 dynamics as if they are a set of new social dynamics and train another CBM as before, like the Level 2 CBM in Fig. 2. Repeating this layer-wise training process for *L* times, we obtain an RCBM of *L* levels. Note that the number of filters *K*_*l*_ at each level can be different, e.g., in Fig. 2, we have *K*_1_ = 3 and *K*_2_ = 2. Also, note that even if the filter scale *T*_*w*_ remains constant across different levels, the higher-level filters will still detect larger-scale dynamics, i.e., a level-*l* filter effectively looks at the dynamics of scale *c* ^*l*−1^ *T*_*w*_. Besides focusing at larger scales, a higher-level filter can also detect the dynamics of higher levels of abstraction, because it is trained using the lower-level activation vectors, which are themselves a non-linear transformation of their input dynamics. This is how RCBM can recognize the compositional structures of social dynamics across different scales and levels of abstractions.

#### RCBM features

While RCBM inherits all the features of CBM (in Section 3.1), it has two additional features that are reflected in its name. First, all levels of an RCBM share the same structure, hence the name “*recursive*”. This ensures that the numbers of activation vectors remain roughly the same across different levels. This is in sharp contrast to other convolutional deep architectures like [10, 12], where the number of activation vectors becomes *K* ^2^ from the second level; this seriously limits the efficiency and scalability of previous algorithms. Second, by using Equation 3, we can decompose the joint probability of the entire RCBM using Bayes’ rule:

$$\begin{array}{c} \begin{array}{ccc} P(X,h) & = & \prod_{l}P\left(X_{l}|h_{l};W_{l},\sigma_{l}\right)P\left(h_{l};\beta_{l}\right)\\ & = & \frac{1}{Z}\exp\left(\sum_{l}\frac{\left|\right|X_{l}-\sum_{k}W_{l,k}⊗h_{l,k}{\left|\right|}_{F}^{2}}{-2\sigma_{l}^{2}}+\frac{\sum_{k}\left|\right|h_{l,k}{\left|\right|}_{1}}{-\beta_{l}}\right), \end{array} \end{array}$$ (12)

hence the name “*Bayesian*”. Moreover, we note that RCBM is normalized locally according to Equation 3. Therefore, the partition function *Z* in Equation 12 can be calculated efficiently using 3 and the first line of Equation 12; this makes various inferences of RCBM efficient. Finally, such a probabilistic formulation also enables many new applications such as conditional inferences and anomaly detection.

#### Model summary

To summarize, RCBM possesses three attractive properties:

- *Good solution quality*: under A1 ∼ A3, RCBM is capable of identifying compositional structures of social dynamics that have provable convergence qualities. This is attributed to our specialized convolution operators (⊗ and ⊙) and stepsize assignment (Equation 9).
- *Efficiency*: the learning of RCBM is efficient and can scale much better than existing convolutional deep learning methods [10, 12]. This is attributed to our specialized convolution operators, stepsize assignment, and the recursive structure.
- *Wide applicability*: RCBM can be applied to a range of applications. For one, it can be used as the feature extractor for supervised tasks. For another, its probabilistic formulation (Equation 12) enables various conditional inferences and anomaly detection.

While all these properties are verified empirically, we formally establish the first two properties in the next section.

## Analysis

We now establish formally that under assumptions A1 ∼ A3, the specialized operators enable the learning of RCBM to produce good solutions efficiently, whereas the conventional one does not. The proof of all theorems in this section is given in Appendix S1.

### Convergence Properties

#### Theorem 1. *Convergence using the proposed convolution*

Suppose a dataset ${\left\{X^{\left(i\right)}\right\}}_{i=1}^{n}$ is generated according to the process in Equation 1 using filters W^*^. Also, suppose Algorithm 1 is used with the stepsize given in Equation 9, where *W*^[0]^ denotes the initial condition and $\hat{W}$ denote the converged solution. Then we have that ∀W^*^,∃*W*^[0]^ s.t.:

$$\begin{array}{c} \hat{W}\overset{p}{\to }W^{*}. \end{array}$$

#### Theorem 2. *Non-convergence using the conventional convolution*

With the same assumptions as in Theorem 1 but supposing that the conventional matrix convolution is used, then ∀W^*^, we have that:

$$\begin{array}{c} \hat{W}\overset{p}{↛}W^{*}. \end{array}$$

The main message from these two theorems is that using the proposed convolution operators can lead to better convergence because it considers the heterogeneity (Assumption A3) in social dynamics. Although, in principle, not every initial condition (*W* ^[0]^) leads to the global optimum since Equation 6 is not jointly convex w.r.t. *W* and *h*. Practically, however, our experimental results (see the Experimental Results section) show that a handful of random initializations suffice to produce good and reliable solutions.

### Runtime Complexity

From Algorithm 1, the bottleneck of training an RCBM is the function optimize_over_h (i.e., Algorithm 1), because it is called repeatedly and that it is itself an iterative algorithm. Accordingly, we break down the runtime complexity analysis into two parts: (1) bounding the number of iterations *r* it takes to solve Algorithm 1, and (2) analyzing the overall runtime complexity while treating *r* as a constant. The first part is established using Theorem 1.

#### Theorem 3. *Required Number of Iterations*

Suppose an accelerated proximal method is used to solve Equation 6 w.r.t. either *W* or *h* but not both. Let *x*^[*r*]^ denote the solution in the *r*-th iteration, x^*^ denote the optimal solution, and *ϵ* denote an error threshold. Then if the stepsizes given in Equation 9 are used, the number of iterations *r* that ensures ||x^*^−*x*^[*r*]^|| ≤ *ϵ* satisfies:

$$\begin{array}{c} r=O(\epsilon^{\frac{-1}{2}}). \end{array}$$

We note that this represents the fastest convergence rate achievable using first-order methods [27], which is attributed to the careful design of stepsizes in Equation 9.

For the second part, both the ⊗ and ⊙ operators take *O*(*DT*_*w*_ *T*) to calculate. Considering *L* levels and *K* filters (and activation vectors) per level, the total complexity is bounded by *O*(*KDT*_*w*_ *TL*). In contrast, previous works [10, 12] use the conventional matrix convolution that requires *O*(*D* ^2^ *T*_*w*_ *T*) per operation. Further, previous approaches need *K* ^2^ activation vectors to calculate from the second level up. Therefore, their total complexity is *O*(*K* ^2^ *D* ^2^ *T*_*w*_ *TL*). Using *K* = 10 and *D* = 10, this represents a huge runtime overhead of two orders of magnitudes.

## Experimental Results

We conduct extensive experiments in the following three directions: (1) the evaluation of RCBM per se, (2) compositional structures in Twitter and Yelp discovered using RCBM, and (3) two new applications enabled by RCBM.

### Dataset Description

#### Twitter

We use the Twitter dataset from [5] that consists of 181M postings during June to December of 2009 from 40.1M users and 1.4B following relationships. To enumerate the information tokens that carry social dynamics (as defined in Section 3.1), in contrast to a few previous authors who use hashtags [5][6], we find the discussion of many interesting events does not include a hashtag. Therefore, we adopt a more general definition using *bursty keywords*, i.e., ones that attract intense attention during a short period of time. We remove common terms (e.g., “the”, “and”, etc.) and apply the classic method in [26] to detect bursty keywords. A total of 0.5M bursty keywords are detected where their corresponding social dynamics are extracted. For better representativeness, we select the dynamics with at least 5 per-min peak usages and 20 total usages around the 30 minutes during their peak times, yielding a 13K-sample dataset of social dynamics.

We characterize each social dynamic using seven features [7] based on the types of users involved and certain graph statistics. For features based on the types of users involved, we consider five types of users. *Initiators* denote the ones who use this keyword before any of his or her friends did. *First-time propagators* and *first-time commentators* denote the users who retweet and tweets, respectively, about this keyword after his or her friends using the same keyword before. *Recurring propagators* and *recurring commentators* denote the users who retweet and tweet, respectively, the same keyword that himself or herself used it before. For graph statistics, we build the evolving graph corresponding to each keyword’s usages, and use the graph’s diamter and the size of the largest connected component LCC, as they are shown to be informative in [7, 30–32]. Later, we will see that all these dimensions provide clear interpretation for the compositional structures found in Twitter (see the Experimental Results section).

#### Yelp

We further use the Yelp dataset from [33] that consists of 1.1M reviews made by 252K users (with 956K friendship edges among them) during the ten-year period from 2004 through 2014. The target of these reviews are 42K businesses in Las Vegas, Phoenix, Edinburgh, Madison, and Waterloo; each of these businesses is considered as an information token. For better representativeness, we select the businesses with at least 40 reviews (i.e. one review per season, on average), yielding a 5.3K dataset of social dynamics. We characterize each social dynamic using six evolving statistics of a business: its numbers of reviews and tips, its average relative rating, the experience (measured by the number of previous reviews) and influence (measured by the number of friends) of the business’s reviewers, and the amount of user responses (that tag each review as useful, funny, or cool). Similarly, these dimensions provide good interpretability to the compositional structures found in Yelp (see the Experimental Results section).

### Evaluation of RCBM

#### Parametric forms

**Fig 3:**
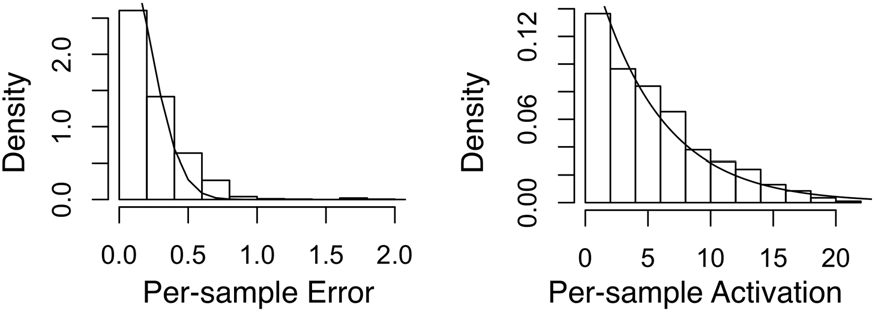
Empirical vs. fitted distributions of error and activation using the Twitter dataset. Left: per-sample error fitted with the Half-Normal distribution; Right: per-sample activation fitted with the Exponential distribution.

We first verify the distributional assumptions we made in Equation 3. To this end, we use each of the two datasets to train a 1-level CBM. For each sample *X*, we calculate the *per-sample error* ||*X*−∑_*k*_ *W*_*k*_⊗*h*_*k*_||_*F*_ and the *per-sample activation* ∑_*k*_||*h*_*k*_||_1_. We then compare their empirical distributions to their model distributions (i.e., according to Equation 3). From the results in Figs. 3 (Twitter) and 4 (Yelp), the empirical distributions and the model distributions seem to match reasonably well. A close examination of the activation vectors confirms that sparsity is enforced effectively such that for each activation vector, most of its elements are exactly zero. These observation supports the validity of our formulations in Equations 3 and 6.

**Fig 4:**
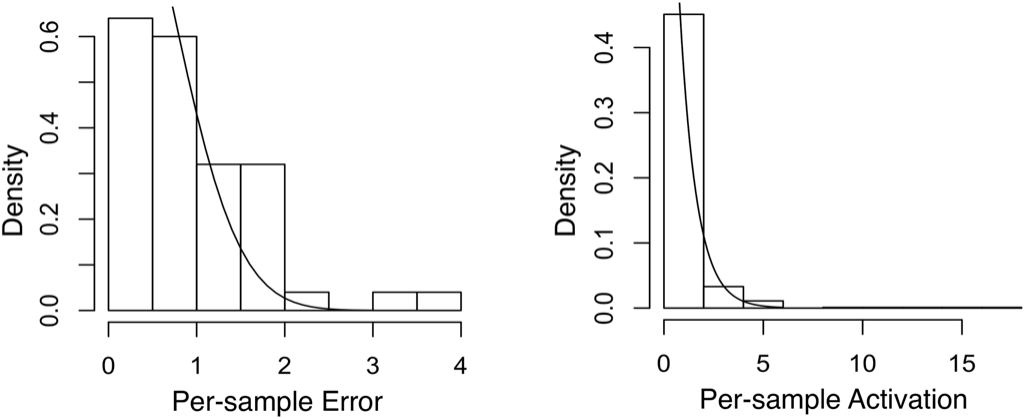
Empirical vs. fitted distributions of error and activation using the Yelp dataset. Left: per-sample error fitted with the Half-Normal distribution; Right: per-sample activation fitted with the Exponential distribution.

#### Runtime and solution quality

We then turn to evaluate the runtime performance and the solution quality of RCBM against deep-learning and non-deep-learning methods. For the baseline deep-learning method, we use cAE [10] as it represents the state-of-the-art convolutional deep learning algorithm. For the proposed method, we test two versions of RCBM: one determines the stepsizes using line search [27] (RCBM-LS); the other uses the proposed fixed stepsize in Equation 9 (RCBM-FS). Using each method, we vary the sample size in the range of 100 to 10000 and train a two-level model with 10 filters at each level. The solution quality of the learnt models is measured using perplexity [29] calculated using 3000 randomly sampled held-out test data. Intuitively, perplexity measures how closely the model distribution resembles the empirical distribution, where a lower value indicates a better model. All experiments are run using 10 repetitions, where both the means and the standard deviations are reported.

**Fig 5:**
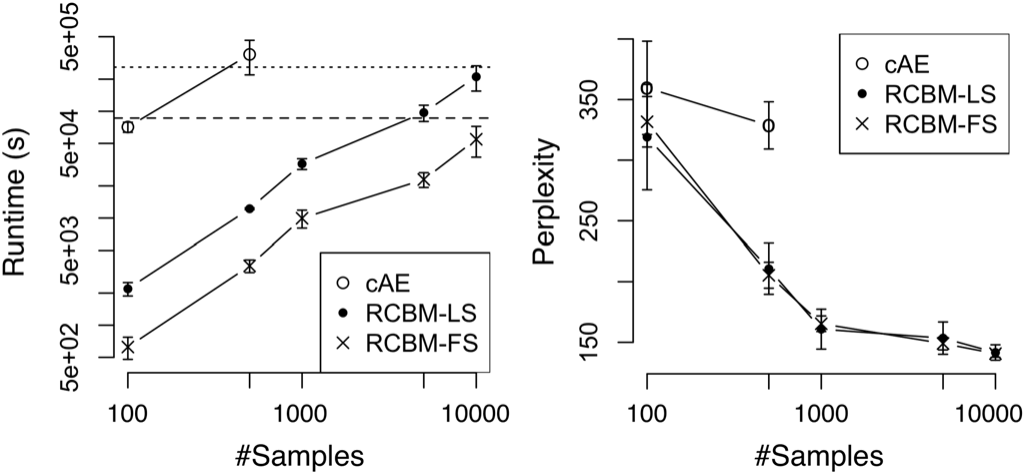
Runtime and perplexity comparisons among deep-learning methods using the Twitter dataset. Baseline: the convolutional autoencoder [10]; LS: RCBM with stepsizes determined by line-search; Proposed: RCBM with stepsizes determined by Equation 9. On the left panel, the dashed and dotted lines mark the runtimes of one and three days, respectively.

From the left panels of Figures 5 (Twitter) and 6 (Yelp), we first observe that RCBM-LS and RCBM-FS run significantly faster than cAE. Indeed, while cAE scales up to 500 samples, both RCBM-LS and RCBM-FS scale to 10,000 samples, which confirms our analysis in Section 4.2. Moreover, RCBM-FS runs much faster than RCBM-LS: while it may take more than 3 days to train an RCBM-LS with 10,000 samples, it takes around 17.5 hours using RCBM-FS. Accordingly, RCBM-FS achieves a 4X ∼ 6X speedup compared to RCBM-LS, or a 30X ∼ 100X speedup compared to cAE. Such a significant speedup is attributed to several of our carefully-designed features, including the fixed stepsizes, the specialized convolutions, and the recursive structure of RCBM.

**Fig 6:**
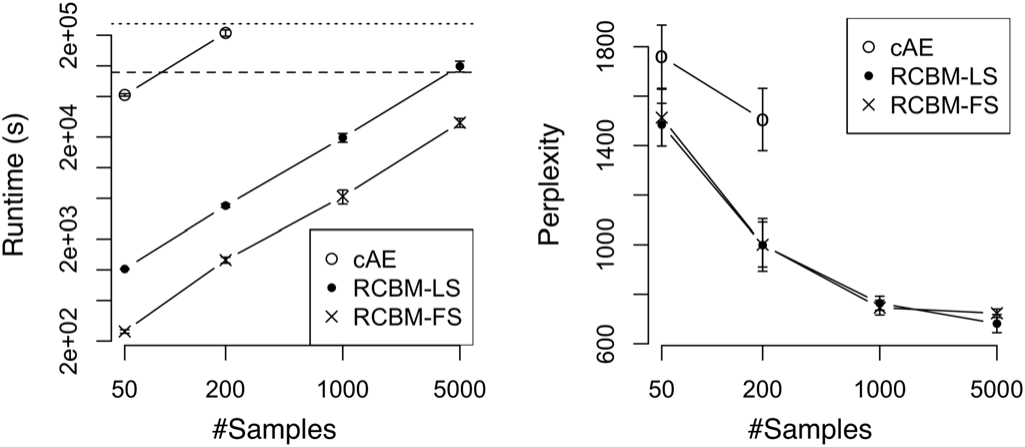
Runtime and perplexity comparisons among deep-learning methods using the Yelp dataset. Baseline: the convolutional autoencoder [10]; LS: RCBM with stepsizes determined by line-search; Proposed: RCBM with stepsizes determined by Equation 9. On the left panel, the dashed and dotted lines mark the runtimes of one and three days, respectively.

For solution quality, we can observe from the right panels of Figs. 5 and 6 that RCBM-LS and RCBM-FS perform comparably and both perform considerably better than cAE, which confirms Theorems 1 and 2. This is because they both incorporate our specialized convolution operators that exploit the heterogeneity of social dynamics, which is not considered by the conventional convolutions used in cAE.

**Fig 7:**
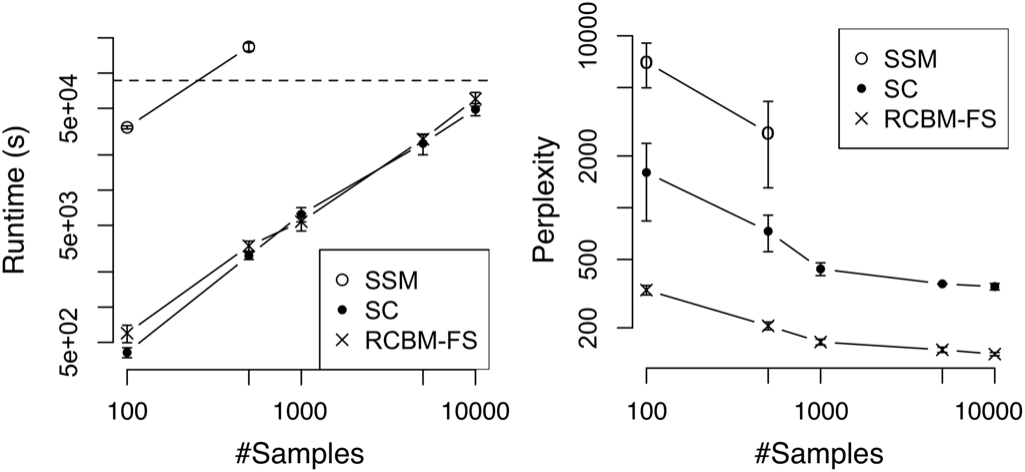
Runtime and perplexity comparisons against non-deep-learning methods using the Twitter dataset. SSM: State Space Model; SC: Sparse Coding; RCBM-FS: the proposed method. On the left panel, the dashed line marks the runtime of one day.

**Fig 8:**
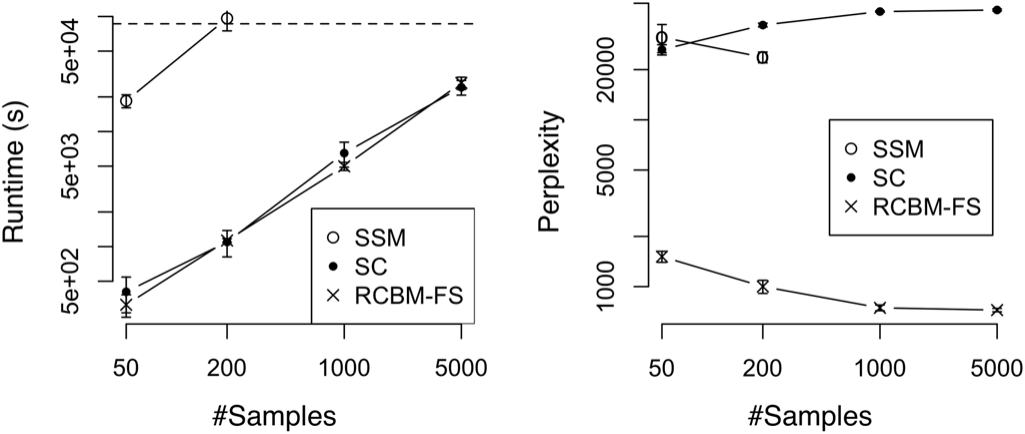
Runtime and perplexity comparisons against non-deep-learning methods using the Yelp dataset. SSM: State Space Model; SC: Sparse Coding; RCBM-FS: the proposed method. On the left panel, the dashed line marks the runtime of one day.

To gain further insight, we compare our proposed method (i.e., RCBM-FS) against two non-deep-learning methods that also incorporate latent factors, i.e., SSM and SC (see Section 2). For a fair comparison, we setup SSM and SC such that each of them has an equal or slightly larger number of parameters compared to that of RCBM-FS. Similar to Figs. 5 and 6, we train these models using the Twitter (Fig. 7) and Yelp datasets (Fig. 8) and present the the runtime and perplexity results.

In terms of runtime (i.e., the left panels of Figs. 7 and 8), we observe that SC and RCBM-FS run much faster than SSM. This is because the standard expectation-maximization (EM) training of SSM involves multiplication and inversion of matrices [20]. Therefore, the complexity for one optimization step is *O*(*n*(*K* ^2^ *T* ^2^+*D* ^2^ *T* ^2^)+*K* ^3^+*D* ^3^), which can be very high for large *n*, *K*, *T*, or *D*. Further, despite that SC is theoretically faster than RCBM by a constant factor *T*_*w*_, we observe that they have comparable runtime in practice. This is attributed to our careful design of stepsize selection, which contributes to a 4 ∼ 6X runtime speedup (see the runtime of RCBM-LS and RCBM-FS in Figs. 5 and 6).

In terms of solution quality, (see the right panels of Figs. 7 and 8), we observe that RCBM-FS performs much better than SSM and SC. The reason why SSM performs poorly is that it makes a rather strong modeling assumption that the dynamical transition of the hidden factors are both linear and time-invariant, which is typically not true in practice. As for SC, the reason is more involved. For the Twitter dataset where the majority of time series have a single peak and are aligned accordingly, SC performs better than SSM because it makes fewer assumptions about the time series dynamics. Still, SC performs poorly compared to RCBM, because it wastes the majority of its parameters in capturing the global signatures at the same scale. In contrast, RCBM-FS uses its parameters more efficiently by exploiting the local signatures of different scales. For the Yelp dataset where the majority of times series have multiple peaks that cannot be aligned, SC performs *worse* than SSM. Indeed, the perplexity even *increases* as the number of samples grows, showing that the incapability of SC to deal with time-shifts represents a serious issue when the time series are not pre-aligned.

#### Efficient selection of *K*

**Fig 9:**
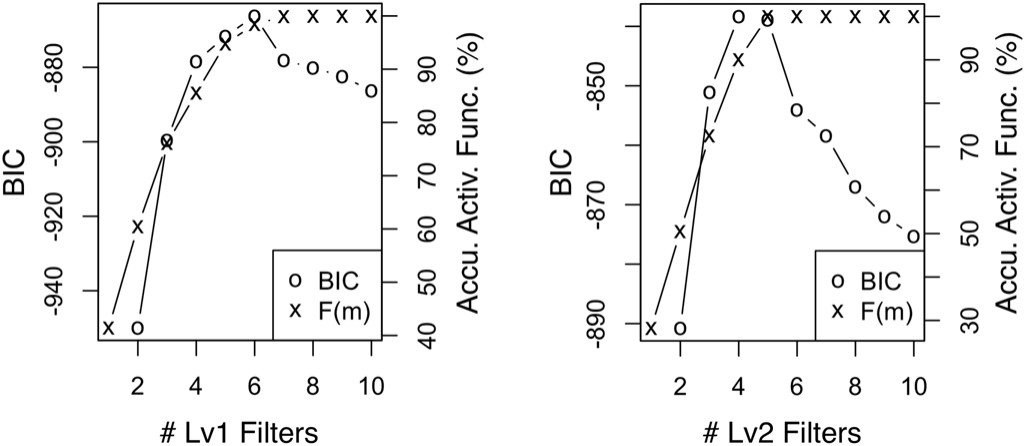
BIC and accumulated activation function of level-1 (left) and level-2 (right) filters using the Twitter dataset. The choices of *K*’s using the two statistics match each other.

Next, we compare the naive and the proposed methods in selecting the best number of filters *K*; both methods are described at the end of Section 3.2. With each of our two datasets, we train two-level RCBM’s with both methods. For the naive method based on Bayesian Information Criterion (BIC), we calculate BIC while fixing *K* = 10 for one of *K*_1_ and *K*_2_ and varying the other; this requires training 20 RCBM’s in total. For the newly proposed method, we train only one RCBM using *K*_1_ = *K*_2_ = 10 while plotting the cumulative activation function *F*(*m*) in Equation 11 for both levels. The results are summarized in Figs. 9 (Twitter) and 10 (Yelp), where good choices of *K* are indicated by peak BIC’s and the points where *F*(*m*) saturates. In both panels of both figures, we observe that the choices of *K*’s suggested by BIC and *F*(*m*) are nearly identical, although it requires training 20 RCBM’s to obtain the BIC curves but only one to obtain the *F*(*m*) curves. Moreover, manual inspection confirms that for the Twitter dataset, *W*_1_ ∼ *W*_5_ of both levels consist of clearly interpretable filters, whereas *W*_6_ ∼ *W*_10_ of both levels consist of plain noise; for the Yelp dataset, similarly, the first six (four) filters in level one (two) are clearly interpretable, whereas the last four (six) filters in level one (two) consist of plain noise. Accordingly, we conclude that the proposed method of choosing *K* is both efficient and effective.

**Fig 10:**
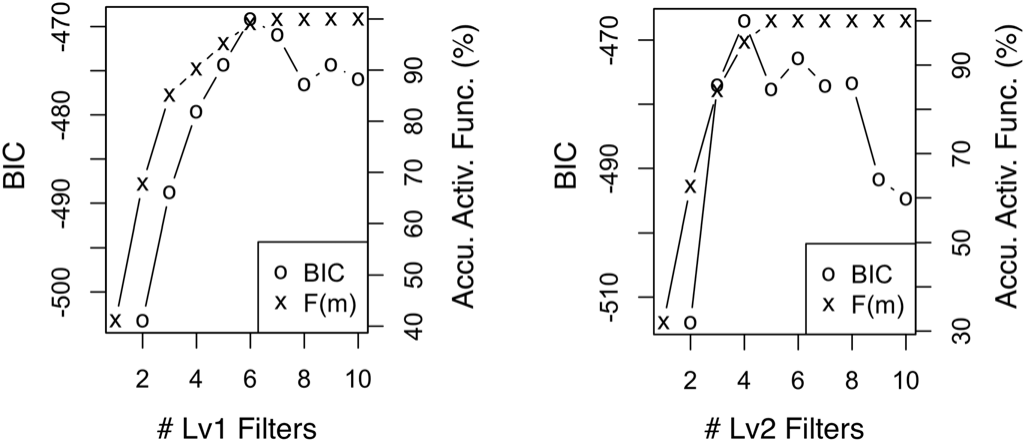
BIC and accumulated activation function of level-1 (left) and level-2 (right) filters using the Yelp dataset. The choices of *K*’s using the two statistics match each other.

### Compositional Structures of Social Dynamics

We now investigate the compositional structures of social dynamics by inspecting the learnt filters (i.e., *W*’s in Equation 12) in RCBM. We first note that this analysis is in sharp contrast with the ones given in [5–7] in two ways. First, the goal in [5–7] is finding representative samples, which is essentially *clustering*; our goal, on the other hand, is finding structures that best characterize social dynamics, which is essentially *decomposition*. Second, our method is compositional and scale-free.

#### Compositional Structures in Twitter

**Fig 11:**
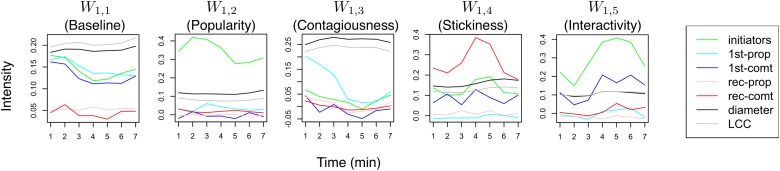
The level-1 compositional structures of Twitter social dynamics identified using a two-level RCBM. They represent the fine-grained signatures of Twitter social dynamics including the baseline, popularity, contagiousness, stickiness, and interactivity.

**Fig 12:**
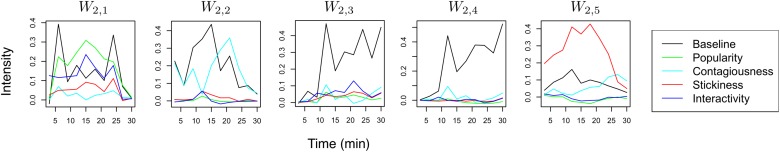
The level-2 compositional structures of Twitter social dynamics identified using a two-level RCBM. They represent the interactions among the fine-grained signatures in Fig. 11.

For the Twitter dataset, we use *K*_1_ = *K*_2_ = 5 according to the experiment in Fig. 9 and train a two-level RCBM. The level-1 filters correspond to compositional structures of seven minutes, whereas the level-2 filters correspond to those of 30 minutes. All these filters are visualized in Figs. 11 and 12. In both figures, the filters are ranked according to their corresponding activation strength (i.e., Equation 10).

#### Level-1 structures

The filter *W*_1,1_ in Fig. 11 represents the baseline of typical Twitter social dynamics. It corresponds to a strong community indicated by the black and grey lines of the graph diameter and LCC (largest connected component), respectively. Such a strong community is mainly attributed to the initiators (green), first-time propagators (light blue), and first-time commentators (blue), but not the recurrent propagators (pink) and commentators (red). Such a baseline structure matches Twitter’s responsive and light-weighted nature. The filter *W*_1,2_ characterizes the *popularity* of social dynamics. It mainly consists of the number of initiators, with minor first-time propagators, characterizing how popular a piece of information is from the external sources outside of Twitter, e.g., TV, web news, etc. The filter *W*_1,3_ characterizes the *contagiousness* of social dynamics that consists of mainly first-time propagators (light blue) and the corresponding strong community indicated by the diameter (black) and the LCC (grey), despite only a small number of initiators (green). The filter *W*_1,4_ characterizes the *stickiness* of social dynamics, which consists of mainly recurrent commentators (red) with smaller but proportional numbers of initiators (green) and first-time commentators (blue). It characterize the capability of a social dynamic to retain the attention of the users and keep commenting about it. We note that the community-related dynamics (diameter and LCC) are also weaker since the corresponding community is much smaller compared to that of *W*_1,3_. The filter *W*_1,5_ characterizes the *interactivity* of social dynamics, which has the largest magnitude of first-time commentators (blue) among all level-1 filters. It characterizes the capability of a social dynamic to motivate users to spend time and comment on it, instead of merely passing it along (i.e., propagating it) to other users.

#### Level-2 structures

We now turn to investigate the level-2 filters as visualized in Fig. 12. Note that each individual component on the right of Fig. 12 corresponds to one level-1 filter in Fig. 11, and that the time scale now is 30 minutes instead of 7 minutes that is the case of Fig. 11. This is because the level-2 filters are intended to capture how the level-1 structures interact with one another and form larger-scale structures with high-level meanings, which is a unique feature of RCBM.

The filter *W*_2,1_ characterizes a three-stage structure that is driven mainly by popularity (the green line), but accompanied by different structures in each of its stages. It is accompanied firstly by contagiousness (light blue), secondly by interactivity (blue) and stickiness (red), and thirdly by combinations of the three. The contagiousness dips in the second stage, but gets enhanced again in the third stage, suggesting that contagiousness *alone* is not enough to sustain long-lasting social dynamics. The filter *W*_2,2_ is mainly composed of strong contagiousness, which dips at around time *t* = 12, and is later continued and enhanced by interactivity and stickiness. Manual inspection shows that the contagiousness results from *reporting* some facts before *t* = 12, whereas it results from *commenting* about the facts, e.g., from famous bloggers, after *t* = 12. The filter *W*_2,3_ and *W*_2,4_ are also driven by contagiousness, but their corresponding contagiousness components have a smaller magnitude. The key difference among the two is that in *W*_2,3_, strong interactivity and stickiness are generated as a result of the initial contagiousness, which is much weaker in the case of *W*_2,4_. As a result, the dynamics with top 10% *W*_2,3_ activations reaches more than three times larger audiences compared to the case of the dynamics with top 10% *W*_2,4_ activations. Finally, the filter *W*_2,5_ exhibits a clear two-stage structure. The second stage characterized by contagiousness (light blue) seems to result from the first stage that is characterized by strong stickiness. Manual inspection shows that such a structure consists of committed core users and responsive peripheral users, which is consistent with the *leader-follower* pattern reported in [7]. In the present work, however, the local structures of the pattern as well as the interaction among these structures are decomposed and analyzed in much greater detail.

To summarize, we find three representative ways where smaller-scale signatures can interact and form lager-scale structures with higher-level meanings. First, popularity can stimulate interactivity, stickiness, and contagiousness (i.e. *W*_2,1_). Second, contagiousness can generate interactivity and stickiness, which, in turn, enhance contagiousness (e.g., *W*_2,2_ and *W*_2,3_). Finally, stickiness beyond a certain threshold can generate contagiousness (e.g., *W*_2,5_).

### Compositional Structures in Yelp

**Fig 13:**
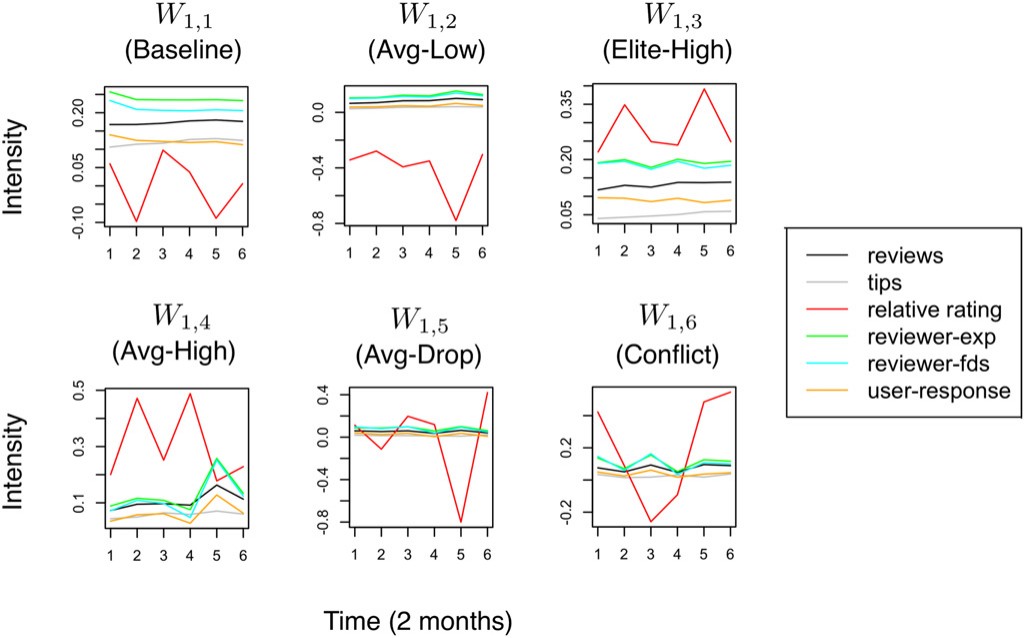
The level-1 compositional structures of Yelp social dynamics identified using a two-level RCBM. They represent the fine-grained signatures of Yelp social dynamics including high and low ratings given from reviewers with different levels of experience.

**Fig 14:**
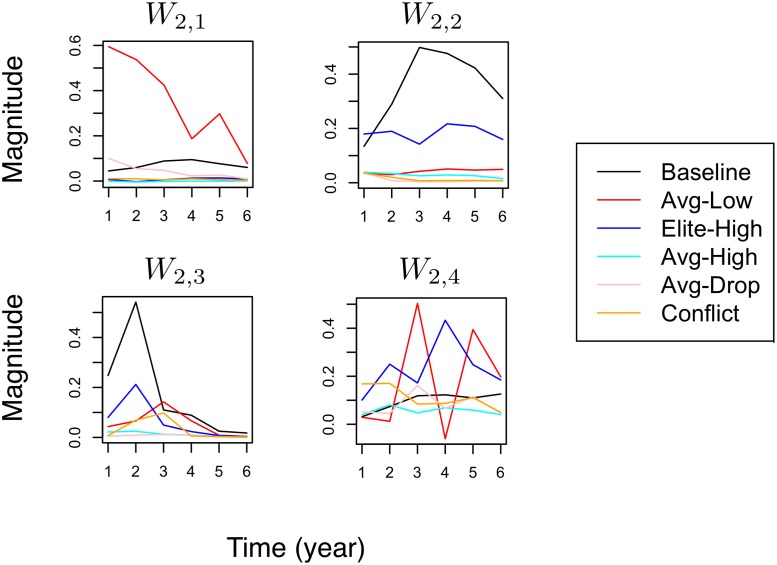
The level-2 compositional structures of Yelp social dynamics identified using a two-level RCBM. They represent the interactions among the fine-grained signatures in Fig. 13.

For the Yelp dataset, we use *K*_1_ = 6,*K*_2_ = 4 according to the experiment in Fig. 10 and train a two-level RCBM. The level-1 filters correspond to compositional structures of one year, whereas the level-2 filters correspond to that of six years. Again, these filters are ranked according to their corresponding activation strength (i.e., Equation 10) and visualized in Figs. 13 and 14.

#### Level-1 structures

Each level-1 structure indicate a particular level of rating (red lines in Fig. 13) given by one of the two types of reviewers: a smaller number of *elite reviewers* who have a higher level of experience (green lines) and influence (blue lines), and a larger number of *average reviewers* who are the opposite of their counterparts. The filter *W*_1,1_ in Fig. 13 represents the baseline of a typical Yelp social dynamic. It corresponds to neutral ratings (indicated by the small magnitude of the red line) given by *elite reviewers* who are more. Since this filter gets activated the most among all level-1 filters, it is consistent with the fact that the majority of Yelp contents are provided by a relatively small set of elite users. Moreover, it is consistent with the fact that most Yelp businesses have ratings close to the overall average (i.e., around 3.7 stars). The filter *W*_1,2_ detect the cases when a business is given low ratings by average reviewers; The filter *W*_1,3_ characterize the case when a business is given high ratings by elite reviewers; The filters *W*_1,4_ and *W*_1,5_ indicate the cases when high and low ratings are given by average users, respectively. Note that there is a difference between *W*_1,5_ and *W*_1,2_: in the former case, the rating for the business was neutral for several months, but drop suddenly at *t* = 5; In the latter case, the rating is low from the beginning, and become particularly so at *t* = 5. Finally, in *W*_1,5_, the rating oscillates significantly, where the extreme values at *t* = 1,3,5 are all driven by elite users. This seem to characterize a conflict in rating a business among different groups of elite users, comparable to the edit wars in Wikipedia [34].

#### Level-2 structures

The level-2 structures of Yelp social dynamics are summarized in Fig. 14. Note that the time scale now is six years instead of one year that is the case of Fig. 13. Again, each individual component on the right of Fig. 14 corresponds to one level-1 filter in Fig. 13. The filters *W*_2,1_ and *W*_2,2_ indicates cases where a business is consistently given low and high ratings by average and elite users, respectively. While it is interesting to see these two common long-term dynamics that present on Yelp, it is equally informative to see that the opposite cases are *uncommon*. That is to say, from our data, it is uncommon to see a business that is consistently given high ratings by average users, or low ratings by elite users. Moreover, the filters *W*_2,3_ and *W*_2,4_ both show disagreement in the ratings of average versus elite reviewers. Indeed, in *W*_2,3_, the high ratings from the elite reviewers (blue line) at *t* = 2 is substituted by the low ratings from the average reviewers (red line) at *t* = 3, accompanied by increased conflict (yellow line) among elite reviewers. Further, the situation becomes more dramatic in *W*_2,4_, where multiple such transitions take place with one-year gaps.

To summarize, we find representative ways where the ratings from the average and the elite Yelp reviewers can interact in different time scales. Particularly, three common long-term structures seem to emerge: (1) low ratings by many average users, (2) high ratings by many elite users, and (3) sharp disagreement and transitions in the ratings between the average versus elite users. The cause and mechanism of these long-term structures are beyond the scope of this work and are left for future research.

### Applications of RCBM

#### Anomaly detection

An advantage of RCBM is its probabilistic formulation (i.e., Equation 12) that assigns a probability to every sample social dynamic. Therefore, a natural application is to detect abnormal social dynamics with extremely-low probabilities. A list of such anomalies detected in Twitter is summarized in Table 2, where examples tweets are listed and the corresponding keywords underlined. Similarly, a list of such anomalies detected in Yelp is summarized in Table 3, where example reviews are listed with their corresponding ratings (i.e., in the parentheses).

**Table 2:** Sample Twitter keywords and tweets that are associated with abnormal social dynamics detected using RCBM. The keywords are underlined.

**Major Disaster**
RT @BreakingNews: BULLETIN—TSUNAMI WARNING ISSUED FOR AMERICAN SAMOA, SAMOA, NIEU, WALLIS-FUTUNA
RT @BreakingNews Tsunami watch iss. for Indonesia, India, Thailand & Malaysia after a powerful 7.9-magnitude earthquake off Sumatra
RT @marcambinder: Breaking: Small plane and helicopter collide over Hudson River in Manhattan. 10–60 (major emergency) declared.
**Urgent message**
RT @SFChron_alert: Obama declares swine flu a national emergency. http://www.sfgate.com/ZILQ
RT from Iran—If you are outside Iran, change your location / timezone to Iran / Tehran to make it harder to track Iranians
#SaveBalloonBoy Colorado Boy Floats Away In Balloon, Frantic Search Under Way To Rescue Boy http://bit.ly/tsxWI
**Major online service shutdown**
And the world has come to an end… Gmail is down.
i hate @youtube for wrongfully banninag @ownagepranks account #youtubefail
**Machine-generated message**
omg!! is it true what they wrote about you in their twit blog? http://lila.twittersblogs.com
EVERYONE!!! Check this new dating site out! Totally Free! talk to mad local chicks that are down for anything! http://local-camz.com
300 new followers in a day—TOTALLY FREE—NO SALE—http://twittertrain.info
Hey everyeone. Just lost 32 lbs in 3 weeks. I wanted to say thanks to Rhonda and her awesome blog. www.rhondasweightloss.com
Hey #JonasOnUstream I LOVE IT (Jonas Brothers live > http://ustre.am/2us4)
CHECK out this site, im a member of it, It gets you more followers: http://TwitTrain.info
Hello!, I just made $842 working a few hours this week from home for Google. You should really check this out! http://bit.ly/u7Rvz
I made an extra $80 today from using tips from http://EARNING-PROFIT3.com
I just took “how sexual are you?” and got: virgin! Try it? http://bit.ly/zM3kl

**Table 3:** Sample Yelp businesses reviews that are associated with abnormal social dynamics detected using RCBM.

**Adult Entertainment**
First time visiting a male-dancing strip club. Never in my wildest dreams did I think I would enjoy this experience as much as I did! (5)
I get accosted and molested by this tall blond Eastern European girl who tried dragging me back to VIP. (1)
I am a strip club aficionado, and this place cannot be beat. (5)
Do you like being crammed into tight spaces and being yelled at by security wherever you stand? (1)
We call it heaven. Real life angels wiggling for our pleasure! (5)
My wife and I dropped $560 here tonight and got almost nothing out of it. (1)
**Poor Service / Facility**
Terrible customer service, hold times are outrageous, issues are rarely fixed in a timely manner. (1)
No stars, but its forcing me to at least do one star to do this review, worst customer service ever!!!! (1)
The store, the people who go there, the parking lot, the area, it is just all gross. (1)
It’s dirty in there, and none of the employees are happy that they have a job. (1)
This mall is sad. You will actually feel bad for this mall. (1)
My son gripped my hand as if we were walking through a haunted house. My wife did the same. (1)
**Consistently Outstanding Restaurants**
The restaurant exceeded our expectation in both food and service. (5)
It is pricey, but the food and service is always consistently excellent. (5)
Loved everything about this place and was surprised it lived up to the hype. (5)
This place was incredible, and totally lived up to the hype. (5)
Thin Crust Pizza at its best. (5)
From start to finish, from wine to dessert and everything in between, this place lived up to all of my expectations and then some. (5)

##### Anomalies in Twitter

The anomalies detected in the Twitter dataset (see Table 2) roughly consist of four groups. The anomalies in the first group correspond to major disasters, including the 2009 tsunamis in American Samoa and Indonesia, and the plane crash in Manhattan. The second group of anomalies corresponds to urgent messages, like the national emergency of swine flu and the 2009 Iran election. The property of the first two groups is that they are very contagious and can form large communities very quickly. However, there is relatively little interaction among users compared to other social dynamics with comparable level of contagiousness. The third group of anomalies corresponds to the shutdown or malfunctioning of major online services like gmail or youtube. The forth group are machine-generated messages, which typically correspond to tweets about some marketing promotion. The last two groups have a common characteristic of having a lot of popularity but barely any contagiousness, stickiness, and interactivity compared to typical social dynamics. Finally, we note that detection of all these four groups of anomalies has useful applications. Indeed, for the first three groups, although they happen rarely, detecting them early and being able to respond to them can have a huge impact. For the last group, it is useful in detecting the online scam.

When analyzing these anomalies, a legitimate question is whether these anomalies can be trivially detected by frequency-based rankings. It turns out that, the list in Table 2 is very *different* from the one generated by ranking keywords using their frequencies. Indeed, only twelve out of top-100 frequent keywords are considered to be associated with anomalies, which is, equivalently, 27 out of top 500 or 46 out of top 1000. To gain further insights, in Table 4, we list some tweets and keywords that are used the most frequently but are associated with normal social dynamics. They can be roughly divided into three groups: holidays, common emotion, and trendy events. It can be observed that each tweet in this table seems to be associated with more organic interactions compared to the cases in Table 2.

**Table 4:** Sample Twitter popular keywords and tweets associated with normal social dynamics.

**Holiday**
Merry Christmas! Anyone staying up to wait for *Santa*?
Happy Thanksgiving to all my friends in the US
**Common emotion**
#VMAs Taylor Swift is amazing. Kanye is so rude. @taylorswift13, you go girl. I’m proud of you:)
RT@newellhj Oh Nick. You are an idiot. This is why you should have been invited. You just show you’re an idiot. #bbcqt
**Trendy events**
Yankees win! Thaaaaaaaaa Yankees win! #WorldSeries #Champs
Hooray! North Korea pardoned the detained US journalists!
So the balloon landed and the little boy isn’t inside?! Where is he?? Ahh!
I wonder if Obama actually wrote this speech because it’s really good.
Hi, i’m Madonna. I’m doing a tribute to Michael Jackson in which i ramble about myself the whole time because i am so very classy.
Watching the emmy’s
Is it wrong that I cried at the glee finale? I wish it was like when I was in high school.

##### Anomalies in Yelp

The anomalies detected in the Yelp dataset (see Table 3) roughly consist of three groups. The anomalies in the first group correspond to adult entertainment businesses. The property of this group is the strong yet distinct preferences from individual reviewers, some calling it “heaven” and giving five starts, while others saying they “got almost nothing out of it” and give only one star. Further, these radically different ratings are mixed uniformly in time, which is in sharp contrast to the transitions that present in *W*_1,6_ of Fig. 13 or in *W*_2,3_ and *W*_2,4_ of Fig. 14 where each transition takes months or years. The second group of anomalies corresponds to exceptionally poor services or facilities for a prolonged period of time. The property of this group is that they consistently receive the lowest possible ratings from both average and experienced reviewers. While long-term negative ratings from average reviewers are common (i.e., see *W*_2,1_ of 14), this group of anomalies get consistent negative ratings from elite reviewers as well. The third group corresponds exclusively to those restaurants that are constantly outstanding. They receive the highest possible ratings frequently, mostly from average users but also from experienced users. Common words that can be found from the reviews of this group of businesses include “consistently excellent”, “lives up the hype”, and “exceeds expectation”. Moreover, unlike the case of *W*_2,2_ of 14 where positive ratings are given by elite users, these business consistently receive top ratings from average reviewers. Finally, these anomalies, again, cannot be trivially detected using frequency-based rankings. Indeed, less then 15% of these anomalies appears in the lists of top-100 businesses in terms of the numbers of reviews, tips, and checkins. This confirms the advantage of RCBM in detecting anomalies according to their social dynamics, which is based on the common compositional structures learnt directly from a large quantity of unlabeled data.

#### Feature extraction for forecasting

When deep learning is used as the unsupervised feature extraction module in Computer Vision and Natural Language Processing [10–14], it produces state-of-the-art results in various supervised learning tasks. Similarly, we explore RCBM’s potential for supervised learning in social applications. For the Twitter dataset, we try to forecast the total number of users of a hashtag; for the Yelp dataset, we aim to forecast the average daily checkins of a business during 2014.

For each dataset, we build a two-level RCBM using a training set. Then, for each testing sample, we obtain its activation vectors using Algorithm 1. To prevent the use of unavailable information during forecasting, for the Twitter dataset, we use all samples up to November 31 as the training set, and all samples in December as the testing set. Also, for each test sample, only the data up to its peak usage time is used. Similarly, for the Yelp dataset, the prediction of the 2014 average checkins are made based on the information up to the end of December 2013. For the prediction models, we use the vector ARMA (VARMA) and Support Vector Regression (SVR) as representative linear and nonlinear models [29], respectively. For features, we use the seven-dimensional features in Fig. 11 as the baseline, and the RCBM activation vectors in the first level (H1), the second level (H2), and in both levels (H1+H2). To gain further insights, we also use another 1-level RCBM with an equal number of parameters as the two-level RCBM (i.e., with doubled number of filters), and use its activation vectors (H1^2^) as features.

The results are summarized in Tables 5 and 6. In general, we observe that SVR performs better than VARMA, whereas using the H1 / H2 / H1+H2 / H1^2^ features also performs much better than using the baseline features. However, an interesting observation is that using the setting VARMA + H1 + H2 performs better than using the setting SVR + Baseline. It suggests that using activation vectors as features can perform reasonably well even when a simple linear model is used. Moreover, it can be observed that using H1 + H2 performs much better than using H1, H2, or H1^2^, no matter whether VARMA or SVR is used. This indicates that exploiting compositional features across different time scales using a multi-layer structure is indeed helpful in forecasting social dynamics. We believe this is an important message with a lot of promising applications, and plan to study it in greater details in our future work.

**Table 5:** Forecasting error (via RMSE) of various models and features using the Twitter dataset. The models used include VARMA and SVR, whereas the features used include the raw social dynamics (Baseline), level-1 activation vectors (H1), level-2 activation vectors (H2), and both levels of activation vectors (H1 + H2) of a 2-level RCBM. The forecasting accuracy using a 1-level RCBM with a doubled number of filters is denoted as (H1^2^).

Method	RMSE
VARMA + Baseline	451.1
VARMA + H1	246.7
VARMA + H2	397.6
VARMA + H1^2^	313.8
VARMA + H1 + H2	**235.9**
SVR + Baseline	397.6
SVR + H1	231.0
SVR + H2	360.4
SVR + H1^2^	281.5
SVR + H1 + H2	**184.2**

**Table 6:** Forecasting error (via RMSE) of various models and features using the Yelp dataset. The models used include VARMA and SVR, whereas the features used include the raw social dynamics (Baseline), level-1 activation vectors (H1), level-2 activation vectors (H2), and both levels of activation vectors (H1 + H2) of a 2-level RCBM. The forecasting accuracy using a 1-level RCBM with a doubled number of filters is denoted as (H1^2^).

Method	RMSE
VARMA + Baseline	892.2
VARMA + H1	647.9
VARMA + H2	674.1
VARMA + H1^2^	655.6
VARMA + H1 + H2	**639.8**
SVR + Baseline	744.5
SVR + H1	584.3
SVR + H2	582.7
SVR + H1^2^	598.1
SVR + H1 + H2	**536.0**

## Conclusion

In this paper, we have introduced a new perspective on studying social dynamics, namely, the multi-scale compositionality. To this end, we have proposed a novel model called RCBM that is capable of both identifying signatures at multiple scales (Task T1) and discovering compositional interactions (Task T2) in social media. Specifically, our contributions are:

1. *Design and Analysis of RCBM*: We have developed RCBM based on specialized convolution operators. While the formulation of RCBM is general enough to consider the heterogeneity of social signals, its runtime performance and solution quality are analyzed formally and confirmed experimentally.
2. *Identifying the Compositional Structures of Social Dynamics*: Using RCBM, we have discovered that the social dynamics in Twitter are characterized by signatures representing the dynamics’ popularity, contagiousness, stickiness, and interactivity. In contrast, the social dynamics in Yelp are characterized by signatures representing how different groups of reviewers rate individual businesses. Moreover, we have found the patterns where theses signatures interact by generating, enhancing, or dominating one another.
3. *RCBM-Enabled Applications*: We have investigated new applications enabled by RCBM, such as detecting abnormal social dynamics and forecasting social dynamics with features learnt using RCBM.

Being the first work that brings deep learning into social networks research, we believe RCBM opens up many opportunities for new research and applications beyond the ones we have demonstrated here. We plan to keep exploring along this direction in our future work.

## Supporting Information

S1 Appendix Proof of Theorem 1, 2, and 3. (PDF)
