# Supplementary material for: Multi-Scale Compositionality: Identifying the Compositional Structures of Social Dynamics Using Deep Learning
Source: PLoS One. 2015 Apr 1;10(4):e0118309. doi: 10.1371/journal.pone.0118309 (PMC4382120; doi:10.1371/journal.pone.0118309)
Supplement: S1 Appendix — (PDF) [file pone.0118309.s001.pdf]

APPENDIX

LEMMA 1. ***Lipschitz Continuity of*** f_1 ***w.r.t.*** W *: Let* $f_1(W,h) = \frac{1}{2}||X^{(i)} - \sum_k W_k \otimes h_k^{(i)}||_F^2$*. Then* $f_1(W,h)$ *is L-Lipschitz-continuous w.r.t.* W_k *with the constant:*

$$L = \sum_i ||h_k^{(i)}||_1^2.$$

PROOF. Using Equation 7 and let $\Delta W_k = W_k - W_k'$, we have:

$$\begin{array}{rl} & ||\nabla f_1(W_k) - \nabla f_1(W_k')|| \\ = & ||\sum_i \tilde{h_k^{(i)}} \otimes h_k^{(i)} \otimes \Delta W_k|| \\ \leq & \sum_i ||\tilde{h_k^{(i)}} \otimes h_k^{(i)} \otimes \Delta W_k|| \\ \leq & \sum_i ||\tilde{h_k^{(i)}}||_1 \cdot ||h_k^{(i)} \otimes \Delta W_k|| \\ \leq & \sum_i ||h_k^{(i)}||_1^2 \cdot ||\Delta W_k|| \end{array} \quad (13)$$

Note that the third line is obtained using triangular inequality, whereas the fourth and the fifth lines are obtained using Young's inequality [32]. □

LEMMA 2. ***Lipschitz Continuity of*** f_1 ***w.r.t.*** h *: Let* $f_1(W,h) = \frac{1}{2}||X^{(i)} - \sum_k W_k \otimes h_k^{(i)}||_F^2$*. Then* $f_1(W,h)$ *is L-Lipschitz-continuous w.r.t.* h_k *with the constant:*

$$L = ||W_k||_1^2.$$

PROOF. Using Equation 7 and let $\Delta h_k = h_k - h_k'$, we have:

$$\begin{array}{rl} & ||\nabla f_1(h_k) - \nabla f_1(h_k')|| \\ = & ||\tilde{W_k} \otimes W_k \otimes \Delta h_k|| \\ \leq & ||W_k||_1 \cdot ||W_k \otimes \Delta h_k|| \\ \leq & ||W_k||_1^2 \cdot ||\Delta h_k|| \end{array} \quad (14)$$

Again, the third and the fourth lines are obtained using Young's inequality [32]. □

PROOF. (of Theorem 1) We will show that $\hat{W} \xrightarrow{p} W^{mle} \xrightarrow{p} W^*$, where W^{mle} denotes the globally optimal solution of Equation 6. First, note that according to Equations 3 and 6, W^{mle} is the maximum likelihood estimator (MLE) of W^*. Since MLE is consistent [30], we have that:

$$W^{mle} \xrightarrow{p} W^*. \quad (15)$$

Now, suppose $W^{[0]} = W^{mle}$ and L denotes that Lipschitz constant given in Lemma 1. Since the stepsize t_W in Algorighm 1 (i.e., Equation 9) satisfies that $t_w < 2/L$, we have that [3]:

$$f(W^{[k]}) - f(W^{mle}) \leq \frac{||W^{[0]} - W^{mle}||^2}{(k+1)^2},$$

where $f(\cdot)$ denotes the objective function of Equation 6. Since that right-hand side (RHS) is zero, trivially, we have:

$$W^{[k]} = \hat{W} \xrightarrow{p} W^*. \quad (16)$$

Combining Equations 15 and 16, we have the proof. □

Remark: Although we use $W^{[0]} = W^{mle}$ in the proof, it can be assigned to any point in the ϵ-ball $B_\epsilon(W^{mle}) = \{W \mid ||W - W^{mle}|| \leq \epsilon\}, \epsilon > 0$, where the objective function is convex in the set $W \in B_\epsilon(W^{mle})$.

Definition 1. **Heterogeneity**: A filter W^* is called *heterogeneous* if $\forall i \neq j$, $\exists \epsilon > 0$ such that:

$$||w_i^* - w_j^*||^2 \geq \epsilon, \quad (17)$$

where w_i^* denotes the i-th row of W^*.

PROOF. (of Theorem 2) We prove a non-trivial special case where the general case can be established similarly. Suppose $D = 2, K = 1$, and h is the delta vector (i.e., one for the element in the middle and zero elsewhere). Let W^{mle} denote the global optimal solution of Equation 6 using the conventional matrix convolution. According to the generation process (that uses the proposed convolution operator) and the definition of the conventional matrix convolution, we have that:

$$\begin{array}{rl} w_1^{mle} = w_2^{mle} \xrightarrow{p} & \arg\min_w ||w - w_1^*||^2 + ||w - w_2^*||^2 \\ & = \frac{1}{2}(w_1^* + w_2^*), \end{array} \quad (18)$$

where w_i^{mle} and w_i^* denote the i-th row of W^{mle} and W^*, respectively. Since W^{mle} is the optimal solution, we have that:

$$||\hat{W} - W^*||^2 \geq ||W^{mle} - W^*||^2, \quad (19)$$

where the RHS converges (in probability) to:

$$\begin{array}{l} 2\left(||\frac{1}{2}(w_1^* + w_2^*) - w_1^*||^2 + ||\frac{1}{2}(w_1^* + w_2^*) - w_2^*||^2\right) \\ = 2(\frac{1}{4}||w_2^* - w_1^*||^2 + \frac{1}{4}||w_1^* - w_2^*||^2) \\ \geq \epsilon > 0. \end{array} \quad (20)$$

Here the last inequality is obtained using Assumption A3 and Equation 17. Using Equations 19 and 20, we have the proof. □

PROOF. (of Theorem 3) Using Lemma 1, Lemma 2, and a convergence result in [19], we know that there is a positive constant c such that:

$$\epsilon = ||x^* - x^{[r]}|| \leq c\, r^{-2}.$$

Equivalently, it follows that:

$$r \leq \sqrt{\frac{1}{c\epsilon}} = O(\epsilon^{\frac{-1}{2}})$$

□
